# The Role and Regulatory Mechanism of Transcription Factor EB in Health and Diseases

**DOI:** 10.3389/fcell.2021.667750

**Published:** 2021-08-13

**Authors:** Sheng-yu Zhu, Ren-qi Yao, Yu-xuan Li, Peng-yue Zhao, Chao Ren, Xiao-hui Du, Yong-ming Yao

**Affiliations:** ^1^Medical Innovation Research Division, Translational Medicine Research Center and Fourth Medical Center of the Chinese PLA General Hospital, Beijing, China; ^2^Department of General Surgery, First Medical Center of Chinese PLA General Hospital, Beijing, China; ^3^School of Medicine, Nankai University, Tianjin, China; ^4^Department of Burn Surgery, Changhai Hospital, Naval Medical University, Shanghai, China

**Keywords:** transcription factor EB, autophagy, tumor, inflammation, organelles

## Abstract

Transcription factor EB (TFEB) is a member of the microphthalmia-associated transcription factor/transcription factor E (MiTF/TFE) family and critically involved in the maintenance of structural integrity and functional balance of multiple cells. In this review, we described the effects of post-transcriptional modifications, including phosphorylation, acetylation, SUMOylation, and ubiquitination, on the subcellular localization and activation of TFEB. The activated TFEB enters into the nucleus and induces the expressions of targeted genes. We then presented the role of TFEB in the biosynthesis of multiple organelles, completion of lysosome-autophagy pathway, metabolism regulation, immune, and inflammatory responses. This review compiles existing knowledge in the understanding of TFEB regulation and function, covering its essential role in response to cellular stress. We further elaborated the involvement of TFEB dysregulation in the pathophysiological process of various diseases, such as the catabolic hyperactivity in tumors, the accumulation of abnormal aggregates in neurodegenerative diseases, and the aberrant host responses in inflammatory diseases. In this review, multiple drugs have also been introduced, which enable regulating the translocation and activation of TFEB, showing beneficial effects in mitigating various disease models. Therefore, TFEB might serve as a potential therapeutic target for human diseases. The limitation of this review is that the mechanism of TFEB-related human diseases mainly focuses on its association with lysosome and autophagy, which needs deep description of other mechanism in diseases progression after getting more advanced information.

## Introduction

Transcription factor EB (TFEB) belongs to the microphthalmia-associated transcription factor/transcription factor E (MiTF/TFE) family, which transcriptionally regulates the expression of multiple genes in response to various stimuli (Napolitano and Ballabio, 2016). Study by Puertollano et al. (2018) showed that the cytoplasmic-nucleus shuttling of TFEB was largely regulated by transitions between phosphorylation and dephosphorylation. In normal condition, TFEB is mainly remained in the cytoplasm with inactivated form mediated by phosphorylation (Martina et al., 2012; Roczniak-Ferguson et al., 2012; Settembre et al., 2012). The activation and translocation of TFEB are sequentially initiated under exposure of cellular stress, such as starvation and lysosomal dysfunction (Martina et al., 2012; Roczniak-Ferguson et al., 2012; Medina et al., 2015). TFEB rapidly shuts down the activation of target genes through nuclear export upon nutrient refeeding (Li et al., 2018a; Napolitano et al., 2018). It has been identified that TFEB is critically involved in the maintenance of structural integrity and functional balance of multiple cells by promoting biosynthesis of lysosomes and mitochondria (Sardiello et al., 2009; Wang S. et al., 2020), which is favorable for the homeostasis of host immune response (Martini-Stoica et al., 2016; Brady et al., 2018b). Activation of TFEB is capable of upregulating the expression of multiple lysosomal proteins by identifying and combining the coordinating lysosomal expression and regulation (CLEAR) elements (Sardiello et al., 2009). By regulating the function and number of lysosomes, TFEB is essential for the completion of all types of autophagy, including macroautophagy, microautophagy, and chaperone mediated autophagy (CMA). Moreover, TFEB is involved in cargo recognition, formation of autophagosomes, and the fusion between autophagosomes and lysosomes (Settembre et al., 2011; Kim et al., 2018). Dysregulation of TFEB activity, however, was reportedly contributed to the development of various diseases, including catabolic hyperactivity in tumors, accumulation of abnormal aggregates in neurodegenerative diseases, and aberrant host responses in inflammatory diseases. Therefore, TFEB might serve as a potential therapeutic target for the treatment of human diseases. For example, the inhibition of TFEB is noted to enhance metabolic pressure or increase chemotherapy sensitivity for tumor cells, while upregulation of TFEB activity is capable of migrating oxidative stress and facilitating the clearance of inflammatory substances in inflammatory states. In terms of neurodegenerative diseases, the accumulation of pathogenic proteins can be alleviated by enhancing TFEB activity due to strong capacity in improving lysosomal function.

## The MiTF/TFE Family of Transcription Factors

In addition to TFEB, other transcription factors have been identified belonging to the MiTF/TFE family, including MiTF, transcription factor EC (TFEC), and transcription factor E3 (TFE3) (Napolitano and Ballabio, 2016; Slade and Pulinilkunnil, 2017). These factors share the similar domains for efficient transcription: the basic domain for DNA binding, and the helix-loop-helix as well as leucine zipper (BHLH-LZ) domains which serve as the formation sites for homodimers or heterodimers in association with the other family members (Aksan and Goding, 1998). Of note, these transcription factors can also form dimers with themselves (Hemesath et al., 1994; Muhle-Goll et al., 1994), which are incapable to bind with other proteins containing BHLH-LZ domains due to unusual three-residue shift within the leucine zipper register (Pogenberg et al., 2012). Unlike other BHLH-Zip transcription factors, MITF/TFE family members can bind the TCATGTG M-box sequence (Napolitano and Ballabio, 2016). Within family members, the domains outside BHLH-LZ are quite different. TFEB has a glutamine-rich domain (Gln) and a proline-rich domain (Pro) (Oppezzo and Rosselli, 2021). MITF and TFEC has a serine-rich domain (Ser), while TFE3 has a proline- and arginine-rich domain (Pro + Arg) (Oppezzo and Rosselli, 2021). In invertebrates, orthologs of MITF/TFE family also exist, including helix-loop-helix transcription factor 30 (HLH-30) in *Caenorhabditis elegans* (Rehli et al., 1999a) and MITF in *Drosophila melanogaster* (Hallsson et al., 2004). They both have basic domains and HLH-zip domains similar to those MITF/TFE family members, and can bind DNA in a similar manner (Bouché et al., 2016).

Results by real-time RT-PCR showed that the MiTF/TFE family was regulated by multiple levels, indicating that these transcription factors might be involved in multiple intracellular processes among disparate tissues (Kuiper et al., 2004). It has been documented that TFEB, TFE3, and MITF share the same regulatory effects in autophagy (Sardiello et al., 2009; Martina et al., 2014; Bouché et al., 2016; Ozturk et al., 2019), which further influence host immune response (Irazoqui, 2020). Indeed, the distinct features of each transcription factor are noteworthy. For example, TFEB is capable of regulating mitochondrial biosynthesis and exerting quality control of mitochondria (Settembre et al., 2013; Nezich et al., 2015; Theeuwes et al., 2020). Meanwhile, MITF is reportedly involved in the development of retinal pigment epithelial and melanocytes (Levy et al., 2006; Ma et al., 2019), and TFE3 is needed for the Golgi stress response (Taniguchi and Yoshida, 2017) and expression of insulin signaling genes (Nakagawa et al., 2006; Iwasaki et al., 2012). However, TFEC appears to be structurally different from the other transcription factors due to lack of acidic domain, thereby functioning as transcriptional inhibition rather than activation (Zhao et al., 1993). TFEC is specifically expressed in bone marrow-derived cells (Rehli et al., 1999b), and is involved in regulating hematopoietic function of hematopoietic stem cells (Mahony et al., 2016).

## The Regulation of TFEB Activity

**FIGURE 1:**
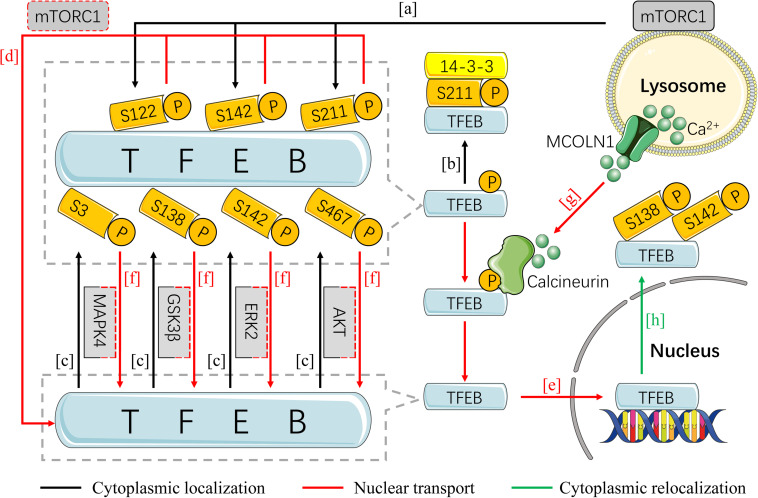
The regulation of TFEB activity by phosphorylation. In the condition of nutritional sufficiency, TFEB is isolated outside the nucleus in an inactive state by phosphorylation and accumulates in the cytoplasm. TFEB can be phosphorylated by mTORC1 on S122, S142, and S211 **(A)**, by AKT on S467, and by GSK3β on S138, by ERK2 on S142, and by MAPK4 on S3 **(C)**. The phosphorylated TFEB on S211 is recognized and bound by YWHA/14-3-3 protein in atypical patterns **(B)**. When encountering with starvation or stress, the withdraw of any phosphorylation site is sufficient to induce TFEB activation and nuclear transport **(D,F)**. Calcineurin is stimulated by the Ca^2+^ released from the lysosome in MCOLN1-dependent way and is primarily responsible for the dephosphorylation of TFEB **(G)**. Dephosphorylated TFEB transfers to nucleus and further initiates the transcription of target genes **(E)**. Refeeding after nutrition deprivation, TFEB initiates cytoplasmic relocalization in a short time by hierarchical phosphorylation on S142 and S138 **(H)**. (AKT, protein kinase B; ERK2, extracellular regulated protein kinase 2; GSK3β, glycogen synthase kinase 3β; MAPK4, mitogen-activated protein kinase 4; mTORC1, mammalian target of rapamycin complex 1; TFEB, transcription factor EB).

A growing body of evidence shows that the transcriptional activity of TFEB is mainly determined by subcellular localization, which largely depends on its phosphorylating status (Puertollano et al., 2018). Under the condition of nutritional sufficiency, TFEB accumulates in the cytoplasm and remains in the form of inactivation by phosphorylation, while it is dephosphorylated and activated in response to starvation, and subsequently transfers to the nucleus for initiating genes transcription. The convert between phosphorylation and dephosphorylation of TFEB and its cytoplasmic-nucleus shuttling are regulated by multiple pathways (Figure 1).

### The Phosphorylation of Cytoplasmic TFEB

The mammalian target of rapamycin complex 1 (mTORC1) is the most widely studied signaling for mediating phosphorylation of TFEB, which is essential for cellular metabolism, growth, and survival via integrating environmental input into downstream pathways (Ardestani et al., 2018). Upon sufficient nutritional supplementation, mTORC1 is recruited to the surface of lysosomes and activated by amino acid and growth factors (Long et al., 2005; Sancak et al., 2010), thereby promoting phosphorylation of TFEB. Up to now, three serine sites have been found responsible for the phosphorylation of TFEB by mTORC1, including S142 (Settembre et al., 2012), S211 (Martina et al., 2012; Roczniak-Ferguson et al., 2012; Settembre et al., 2012), and recently discovered S122 (Vega-Rubin-de-Celis et al., 2017; Figure 1A). It has been demonstrated that the inactive form of TFEB is maintained mainly by phosphorylation of S142 and S211 (Martina et al., 2012; Roczniak-Ferguson et al., 2012), while the phosphorylation of TFEB on S211 is recognized and bound by YWHA/14-3-3 protein in atypical patterns, which inhibits its nuclear translocation (Martina et al., 2012; Xu et al., 2019; Figure 1B).

In addition to mTORC1, TFEB is noticed to be phosphorylated and inhibited by other factors, including protein kinase B (AKT) on phosphorylation of S467 (Palmieri et al., 2017b), glycogen synthase kinase 3β (GSK3β) on S138 (Li Y. et al., 2016), extracellular regulated protein kinase 2 (ERK2) on S142, and mitogen-activated protein kinase 4 (MAPK4) on S3 (Hsu et al., 2018; Figure 1C). Nevertheless, their relationship with cytoplasmic retention of TFEB remains largely unknown.

### Nuclear Transfer of TFEB by Dephosphorylation

The covert between phosphorylation and dephosphorylation status is the determinant for TFEB activation and nuclear transfer. In the setting of starvation, mTORC1 dissociates from the lysosome due to inactivation, which further induces the dephosphorylation of TFEB (Figure 1D). The latter subsequently enters the nucleus for augmenting transcriptional activity of targeted genes followed with dissociation from YWHA/14-3-3 (Martina et al., 2012; Roczniak-Ferguson et al., 2012; Figure 1E). Similarly, withdraw of AKT, GSK3β by the activation of protein kinase C (PKC), ERK2, and MAP4K3 on TFEB phosphorylation is sufficient to initiate TFEB activation and nuclear translocation (Settembre et al., 2011; Li Y. et al., 2016; Palmieri et al., 2017b; Hsu et al., 2018; Figure 1F). In addition, activation of AMP-activated protein kinase (AMPK) promotes the activation of TFEB through inhibiting mTORC1 pathway (van Veelen et al., 2011; Brady et al., 2018a). AMPK can also upregulate dephosphorylation and nuclear localization of TFEB independently of mTORC1 (Collodet et al., 2019). And pharmacological activation of AMPK or inducing loss of FLCN, the negative regulator of AMPK, will inhibit this progress (El-Houjeiri et al., 2019).

Calcineurin is thought to be responsible for the dephosphorylation of TFEB, which can be activated by lysosomal Ca^2+^ (Medina et al., 2015; Figure 1G). Endoplasmic reticulum stress and reactive oxygen species (ROS) can promote the effects of calcineurin on TFEB directly or indirectly through mucolipin 1 (MCOLN1), a lysosomal Ca^2+^ channel (Martina et al., 2016; Zhang et al., 2016). However, a recent study has shown that proteasome inhibition leads to a significant increase in TFEB dephosphorylation level and nuclear translocation (Li et al., 2019), hinting critical involvement of ubiquitin proteasome pathway on TFEB activation, which needs further elucidation on precise mechanism and discrepancy across disparate studies.

### Nuclear Export of TFEB by Re-phosphorylation

It has been clarified that the intracellular localization of TFEB is the determinant of its activation, namely shuttle between cytoplasm and nucleus. Likewise, delayed or disturbed exportation of nuclear TFEB might pose threat to the intracellular homeostasis. Two recent studies have shown that refeeding after nutrition deprivation results in cytoplasmic relocalization of TFEB in a short time through inducing hierarchical phosphorylation of S142 and S138, suggesting that re-phosphorylation of nuclear TFEB is the major mechanism for its cytoplasmic relocalization (Li et al., 2018a; Napolitano et al., 2018; Figure 1H). Study conducted by Napolitano et al. (2018) also identified a highly evolutionary conserved sequence in the N-terminal of TFEB, named nuclear export signal (NES) due to its indispensable role in the cytoplasmic relocalization of TFEB, which was largely impaired by NES mutation. Intriguingly, both the S142 and S138 are located near NES (Napolitano et al., 2018), implying the possible role for NES on the re-phosphorylation of TFEB at both sites. Notably, it has been indicated that inhibition of XPO1, a nuclear export protein, can facilitate the nuclear localization of TFEB without affecting mTOR activity (Silvestrini et al., 2018). However, study that deals with nuclear export of TFEB is still rare, let alone its specific mechanism.

### Other Post-transcriptional Modifications of TFEB

In addition to phosphorylation, other post-transcriptional modifications are also involved in the regulation of TFEB. For instance, the acetylation of TFEB at K274 and K279 affects its transcriptional activity by disrupting dimerization and inhibiting binding to promoters (Wang Y. et al., 2020), which can be reversed by deacetylation (Bao et al., 2016). The SUMOylation of TFEB at a lysine site will lead to decreased transcriptional activity (Miller et al., 2005), while the ubiquitination and further degradation of phosphorylated TFEB will facilitate TFEB activation *via* ubiquitin-proteasome pathway (Sha et al., 2017).

### The Self-Regulation of TFEB

**FIGURE 2:**
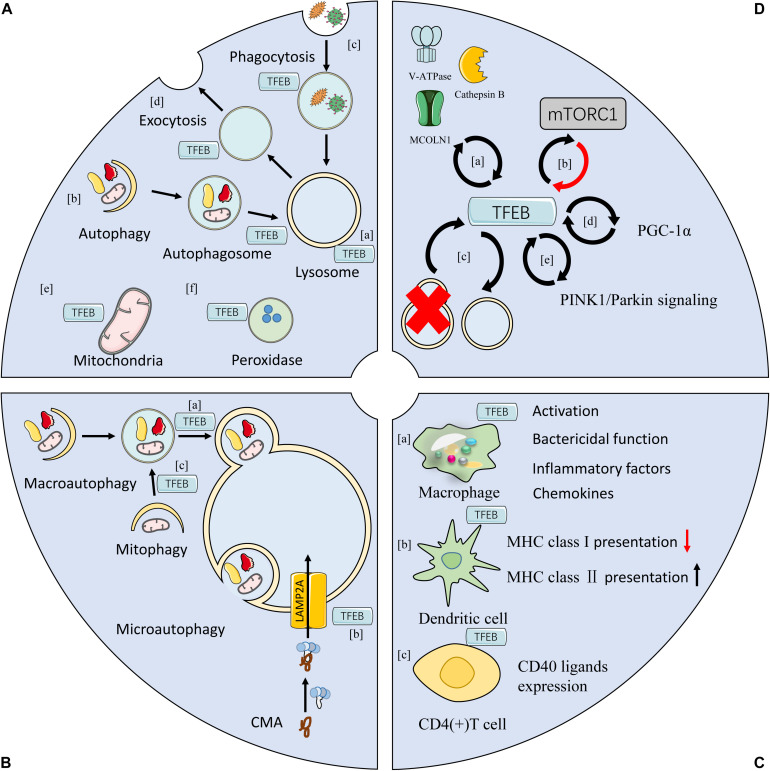
Functions of TFEB. **(A)** TFEB is involved in the biogenesis of lysosomes (a), mitochondrial (e), peroxidases (f), and autophagosomes (b). **(B)** TFEB participates in multiple types of autophagy, including macroautophagy, microautophagy, and chaperone mediated autophagym (CMA) by regulating the function of lysosomes. It is also involved in autophagy at various steps, such as the fusion of autophagosome with lysosome in macroautophagy (a), the localization and trafficking of LAMP2A in CMA (b), and the priming the clearance of damaged mitochondrial in mitophagy (c). **(C)** TFEB regulates immune responses in both innate and adaptive immune systems. TFEB is involved in activation, bactericidal capacity, and production of inflammatory mediators in macrophages (a). In dendritic cells, TFEB inhibits the antigen presentation of MHC class I but enhances the expression of MHC class II (b). In T cells, TFEB is related to antibody response in reaction to the activation of CD40 at cellular surface (c). **(D)** The modulation of TFEB activity. Lysosomal proteins, such as V-ATPase, cathepsin B, and MCOLN1 that are induced by TFEB, can regulate TFEB activity in return (a). mTORC1 is critically involved in the TFEB phosphorylation, which can be activated by TFEB (b). TFEB is a major regulator of ALP, and both lysosomal damage and autophagy disruption result in TFEB activation (c). TFEB directly activate PGC-1α, and it can be activated in a PGC-1α-dependent manner (d). TFEB induces mitophagy by activating PINK1/Parkin pathway, and its activity is also enhanced by stimulating PINK1/Parkin (e). (mTORC1, mammalian target of rapamycin complex 1; CMA, chaperone mediated autophagy; PGC-1α, peroxisome proliferator-activated receptor γ coactivator 1α; TFEB, transcription factor EB; MHC, major histocompatibility complex).

Interestingly, targeted molecules of TFEB are in turn involved in regulating TFEB activity, implicating the self-regulation of TFEB (Figure 2). For example, MCOLN1 is responsible for TFEB dephosphorylation by inducing the release of Ca^2+^ from lysosomes (Medina et al., 2015; Figure 2Da). Cathepsin B, a subtype of hydrolase located within the lysosomal lumen, down-regulates the activation of TFEB by degrading MCOLN1 (Man and Kanneganti, 2016; Figure 2Da). V-ATPase, the H^+^ pump on lysosomal membrane, can activate TFEB nuclear translocation after disrupting its catalytic subunit A (Kim D. et al., 2017; Figure 2Da). TFEB-mediated endocytosis enhances the assembling and activation of mTORC1, which is closely relatedto TFEB phosphorylation on lysosomal membranes through prompting the formation of endosomes (Nnah et al., 2019; Figure 2Db). TFEB is indeed essential for the autophagic process, while its activation is also commonly evident during lysosomal damage or autophagy disruption (Settembre et al., 2011; Roczniak-Ferguson et al., 2012; Figure 2Dc). Moreover, TFEB directly activates peroxisome proliferator-activated receptor γ coactivator 1α (PGC-1α), and it can be activated in a PGC-1-dependent manner (Tsunemi et al., 2012; Kim Y.S. et al., 2017; Erlich et al., 2018; Figure 2Dd). Notably, it was revealed that knockout of either TFEB or PGC-1 resulted in decreased expression of the other gene (Scott et al., 2014). In addition, TFEB was found to be associated with the activation of mitophagy by regulating PTEN-induced putative kinase 1 (PINK1)/Parkin signaling pathway, and its expression was also influenced by PINK1/Parkin signaling (Ivankovic et al., 2016; Figure 2De).

## The Function of TFEB

Activated TFEB can transfer to nucleus and initiates transcription of various genes. TFEB contributes to the maintenance of cellular structure and function via promoting the biosynthesis of multiple organelles and autophagic activities. TFEB also plays a role in host defense machinery by regulating host immune response.

### The Effects of TFEB on Biosynthesis and Function of Multiple Organelles

Lysosome serves as the digestive center of eukaryotic cells, and it is programmed to recycle and degrade biomacromolecule by means of phagocytosis, endocytosis, and autophagy, thereby providing raw materials for cellular anabolism. Meanwhile, it can act as a signaling hub accounting for the main regulatory mechanism of nutrition sensing (Lawrence and Zoncu, 2019). Activation of TFEB is capable of upregulating the expression of multiple lysosomal proteins by identifying and combining the CLEAR elements, which is the promoter region of multiple lysosomal genes (Sardiello et al., 2009). Studies have been demonstrated that TFEB not only regulates lysosomal biosynthesis (Figure 2Aa), but enhances the expression of multiple genes governing lysosomal related processes, including autophagy, phagocytosis, and exocytosis (Palmieri et al., 2011; Figures 2Ab–d). For example, activation of TFEB was identified to augment the bactericidal performance of macrophages *via* promoting its phagocytic activity (Gray et al., 2016). TFEB was found to facilitate lysosomal fusion with the plasma membrane and exocytosis by triggering activation of MCOLN1 in Ca^2+^ dependent mechanism (Medina et al., 2011). Furthermore, TFEB was reportedly capable of regulating the location of lysosomes by increasing TMEM55B expression (Willett et al., 2017).

Mitochondria is the intracellular “energy factory” which plays an important role in host immunity, inflammation, and metabolism (Spinelli and Haigis, 2018; Breda et al., 2019; Vringer and Tait, 2019). TFEB is involved in mitochondrial biosynthesis by inducing the expression of PGC-1α, the major factor for regulating mitochondrial biosynthesis (Ma et al., 2015; Erlich et al., 2018; Figure 2Ae). TFEB mediates the increased expression of multiple mitochondrial proteins in PGC-1α independent manners. For example, mitochondrial transcription factor A (TFAM) is a key factor in directly regulating mtDNA transcription and can be activated by nuclear respiratory factor (NRF)1 and NRF2 (Farge and Falkenberg, 2019). In skeletal muscle of PGC-1α knockout mice, overexpression of TFEB was noted to upregulate the expression of NRF1, NRF2, and TFAM, and mitochondrial volume as well as density simultaneously (Mansueto et al., 2017). In addition, TFEB is essential for mitochondrial stability in mitochondrial dynamics through reducing mitochondrial division (Santin et al., 2016). Functionally, activation of TFEB optimizes mitochondrial substrate utilization, which is favorable for respiratory function and accelerates the production of ATP (Mansueto et al., 2017; Zhang et al., 2019), while it may lead to the accumulation of dysfunctional mitochondria when inhibiting TFEB activity (Zhang et al., 2019).

Recently, TFEB was found to be capable of controlling the biogenesis of peroxidases at the transcriptional level (Figure 2Af), as shown by significantly reduced levels of peroxidases due to inhibition of TFEB (Eun et al., 2018). Furthermore, inhibition of mTORC1 was reported to alleviate the abnormal biogenesis of peroxidases (Eun et al., 2018), which could be partly attributed to the counteract of TFEB inhibition. Additionally, TFEB promoted the biosynthesis of autophagosomes and its subsequent fusion with lysosomes (Settembre et al., 2011).

### Regulatory Impacts on Autophagy

Autophagy refers to a tight regulated process that transports a variety of cellular components to lysosomes for degradation, which involves the renewal of multiple organelles and the removal of misfolded proteins and even invaded intracellular pathogens. Strikingly, TFEB is essential for the completion of all types of autophagy, including macroautophagy, microautophagy, and CMA, by regulating the function and number of lysosomes.

In fact, TFEB has been confirmed involving in multiple steps of autophagic process. For example, macroautophagy, deemed as a highly conserved catabolic process, is clarified to be comprised of various steps, including formation of autophagosomes and further fusion with lysosomes, as well as cargos degradation (Yu et al., 2018). TFEB is required for the biosynthesis of autophagosomes and its fusion with lysosomes (Settembre et al., 2011; Figure 2Ba). CMA is completed by transporting and degrading target proteins in a molecular chaperone dependent manner (Kaushik and Cuervo, 2018). Lysosome-associated membrane protein 2A (LAMP2A) is the firstly identified lysosomal component for the completion of CMA. Of note, activation of TFEB enhances CMA by facilitating the trafficking and localization of LAMP2A (Zhang et al., 2017; Figure 2Bb). Similarly, TFEB participates in identifying cargos during the initiation of selective autophagy. Taking mitophagy as an example, one of its priming mechanism is PINK1/Parkin signaling pathway, which is reportedly TFEB-dependent (Kim et al., 2018; Figure 2Bc). Activation of TFEB can promote the recruitment of autophagosomes to the surface of mitochondria (Tan et al., 2019b).

### Immune and Inflammatory Responses

TFEB is involved in host response by regulating both innate and adaptive immunity (Brady et al., 2018b; Irazoqui, 2020). It has been documented that TFEB participates in the process of macrophage activation by following mechanisms (Fang L. et al., 2017), including promoting bactericidal function (Gray et al., 2016; Liu X. et al., 2019) and facilitating the production of various proinflammatory mediators (Visvikis et al., 2014; Pastore et al., 2016; Figure 2Ca). TFEB is demonstrated to play a role in innate immune response by modulating xenophagy, a form of selective autophagy targeting invaded microorganisms (Wang and Li, 2020). For example, persistent activation of nuclear TFEB resulted in the scavenging activity of functional lysosomes, which further suppressed the replication of *Salmonellas via* targeting *Salmonella*-containing vacuoles (Ammanathan et al., 2019). Moreover, TFEB was essential for the function of dendritic cells (DCs) when encountering with different exogenous antigens (Figure 2Cb). It has been identified that activation of TFEB inhibits the antigen-presentation capacity of major histocompatibility complex (MHC) class I, but upregulates the expression of MHC class II (Samie and Cresswell, 2015). Likely, TFEB is critically involved in T-cell-dependent antibiotic activity by cooperating with TFE3 in response to the activation of CD40 on the surface of T cells (Huan et al., 2006; Figure 2Cc). In addition, TFEB functions as a proinflammatory mediator by promoting the lysosomal degradation of anti-inflammatory cytokines (He et al., 2011).

As a critical component of immune and inflammatory responses is regulated by TFEB. TFEB can be activated by Ca^2+^ signaling to induce lysosomal endocytosis and further promote IL-1β secretion in human monocytes (Tseng et al., 2017). It also collaborates with TFE3 to induce gene transcription of proinflammatory cytokines in activated macrophages and microglia (Pastore et al., 2016). While in TFEB and TFE3 deficient cells, the secretion of key inflammatory mediators, including CSF2, IL1β, IL2, and IL27, was significantly suppressed (Pastore et al., 2016), supporting the role of TFEB in regulating inflammatory response. Moreover, TFEB can induce the expression of peroxisome proliferator activator receptor α (PPAR-α) and PGC-1α to promote mitochondrial biogenesis and thus indirectly reduce ROS production and inflammation (Kim Y.S. et al., 2017).

In addition, TFEB has been found to play a role in controlling metabolism including lipid, energy, amino acid and glucose (Pastore et al., 2017). TFEB takes a part in the metabolism of lipid by regulating lipid transport and lipophagy (Zechner et al., 2012), while overexpression of TFEB perturbs expressions of numerous genes covering fatty acid-binding, transport and oxidation (Settembre et al., 2013). What’ s more, transcriptome analysis in murine cardiomyocytes with TFEB depletion revealed that TFEB regulates a gene network involved in lipid and carbohydrate metabolism independently of macroautophagy (Trivedi et al., 2020). TFEB also responds to the disturbance in energy balance. The increased energy demand during exercise can provoke TFEB expression and activation to improve energy metabolism through mitochondrial biogenesis and mitophagy (Erlich et al., 2018). The cellular response to amino acid signal is primarily via RAGs, heterodimers formed by the combination of RagA/B and RagC/D (Zhu et al., 2020). It has been demonstrated that defective mTOR-TFEB signaling is involved in the progression of RagC^*S*75Y^ mutated cardiomyopathy, which can only be corrected by overexpression of TFEB but not inhibition of mTOR (Kim et al., 2021). Therefore, TFEB may play a role in amino acid metabolism by regulating RagC and need further study to verify. As for glucose metabolism, TFEB can control the expressions of glucose transporters, glycolytic enzymes and glucose homeostasis pathways (Mansueto et al., 2017). And glucose uptake can be increased by TFEB in ways of activating AKT and upregulating insulin receptor substrate 1 (IRS1) and IRS2 (Sun et al., 2021).

## The Role of TFEB in Human Diseases

Since TFEB is essential for maintaining the stability of cellular structure and function, the dysregulation of TFEB activity appears to be associated with the occurrence and development of various human diseases, such as tumors, neurodegenerative diseases, and inflammatory diseases, implying the modulation of TFEB a potential therapeutic target (Table 1).

**TABLE 1:** TFEB serves as a potential therapeutic target for human diseases.

**Diseases**		**TFEB as a disease inductor**	**TFEB as a treatment target**
Tumors	Pancreatic cancer	Increased TFEB promotes autophagy to meet the metabolic needs for growth and metastasis of tumor cells	Inhibition of TFEB enhances the sensitivity to chemotherapy
	Lung cancer	Upregulated TFEB enhances the release of cathepsins to facilitate metastasis; Increased TFEB develops resistance to chemotherapy	Down-regulation of TFEB augments the sensitivity to chemotherapy
	Breast cancer	Activated DNA repair inhibits apoptosis of tumor cells	Inhibition of TFEB in breast cancer cells promotes tumor cell death; upregulated TFEB in macrophages enhances the inhibition of tumor progression
	TFEB RCC	Translocation or rearrangement of TFEB gene leads to nuclear TFEB overexpression	−
Inflammatory diseases	Atherosclerosis	−	Antioxidation in endothelial cells; the enhancement of lysosomal function in macrophages; lipophagy promotion in vascular smooth muscle cells.
	Sepsis	−	Autophagy restoration by upregulation of TFEB
	Osteoarthritis	Inhibited expression and nuclear import of TFEB	Autophagy promotion by TFEB overexpression
Neurodege nerative diseases	Parkinson disease	Cytoplasmic retention of TFEB leads to decreased lysosomal function and excessive α-synuclein production	Activation of TFEB reverses lysosomal dysfunction and reduces oxidative stress
	Alzheimer disease	−	Elevated TFEB expression or deacetylation of TFEB improves the degradation of tau
	Huntington disease	Reduced TFEB level and target genes expression	Activation or overexpression of TFEB increases the clearance of HTT

### Tumors

Hyperactive catabolism is the underlying mechanism for the uncontrolled growth and metastasis of the tumor. It has been reported that TFEB may contribute to the development and progression of tumor by upregulating autophagy and endocytosis to meet the metabolic requirement in pancreatic and prostate cancer cells (Perera et al., 2015; Blessing et al., 2017). TFEB can inhibit the apoptosis of breast cancer cells by activating DNA repair (Slade et al., 2020). And the tumor progression of breast cancer can be inhibited by macrophage-specific TFEB overexpression through enhancing the functional status of immune cells within the tumor microenvironment, as inhibition of TFEB in macrophages obviously leads to the growth of breast cancer (Fang L. et al., 2017). TFEB also mediates tumor metastasis. For example, in a mouse model of lung cancer with liver metastasis, TFEB augmented the metastasis by increasing autophagy through upregulating cathepsins secretion and lysosome synthesis (Kundu et al., 2018). In fact, the TFEB shows divergent impacts on the treatments of various kinds of cancers. With regard to the melanoma and multiple myeloma, for example, elevated expression of TFEB presented anticancer effects *via* triggering autophagic cell death (Cea et al., 2013; Silvente-Poirot et al., 2018). In breast cancer, however, the partial functional loss of TFEB by the interaction of signal transducer and activator of transcription 3 (STAT3) enhances lysosomal-mediated cell death, thus serving as an anticancer action (Li et al., 2018b). In glioblastoma, inhibition of TFEB resulted in the death of tumor cells due to its incapacity of withstanding growing metabolic pressure (Sung et al., 2019). TFEB is found to be involved in chemoresistance in cancer. For instance, TFEB reduced the sensitivity of LoVo and HeLa cells to doxorubicin by inducing activation of autophagy (Fang L. M. et al., 2017), and it reduced the apoptosis of breast cancer cells in response to doxorubicin by repairing DNA damage (Slade et al., 2020). In addition, increased TFEB expression was noticed in both glioblastoma and lung cancer developing resistance to chemotherapy (Karagounis et al., 2016; Sung et al., 2019). Given that, inhibition of TFEB by gene silencing or drugs can markedly enhance the sensitivity of tumor cells to chemotherapy among glioblastoma, lung cancer, pancreatic cancer, and ovarian cancer through either impairing autophagy flux or inducing mitochondrial apoptosis (Karagounis et al., 2016; He et al., 2018; Mitrakas et al., 2018; Song et al., 2019). Therefore, exploring anticancer strategies underlying regulation of TFEB should be based on distinct tumor types.

Recently, TFEB was noticed to be closely associated with the development of t(6;11) renal cell carcinoma (RCC), an extremely rare subtype that occurred in adolescents and accounted for 0.02% of all kidney cancers (Inamura, 2017). Since t(6;11) translocation fuses the TFEB gene on chromosome 6 with the metastasis associated lung adenocarcinoma transcript 1 (MALAT1) gene on chromosome 11, the rearrangement of the fused gene consequently retains the entire TFEB coding sequence and further induces the promoter activity of MALAT1, thereby resulting in the overexpression of nuclear TFEB (Zhan et al., 2018; Caliò et al., 2019). Another phenotype of TFEB-related RCC named TFEB-amplified RCC is independent of TFEB rearrangement, which has been identified sharing feature of nuclear TFEB overexpression similar to t(6;11) RCC (Caliò et al., 2019), but occurring in older patients with a more aggressive clinical course (Argani et al., 2016). Currently, the mechanism underlying RCC development by TFEB overexpression remains unclear. The mouse model of renal carcinoma due to specific renal TFEB overexpression was established, and it was found that highly activated Wnt pathway played an important role in TFEB-induced RCC. Antagonizing Wnt pathway could restrain the proliferation of cancer cells and relieved or even completely reversed the pathological phenotype of RCC (Calcagnì et al., 2016).

### Inflammatory Diseases

Dysregulation of TFEB leads to the imbalance of inflammatory response, which is one of the major causes for inflammatory diseases. TFEB has been shown to play a protective role in endothelial cells *via* migrating oxidative stress and upregulating the expression of multiple antioxidant genes, thereby slowing down the progression of atherosclerosis (Lu et al., 2017). As the atherosclerosis progressed, macrophages are recruited into the growing plaques for the clearance of deposited lipids and apoptotic cells. While TFEB can enhance the lysosomal function in macrophages and prompt the phenotypic shift of macrophages to anti-inflammation subtype, thereby reducing the burden on atherosclerotic plaques (Sergin et al., 2017; Evans et al., 2018; You et al., 2020). While Stearyl coenzyme A desaturase 1 (SCD1) can promote TFEB-mediated lipophagy to interfere with foam cell formation and modulation of the SCD1/TFEB machinery may offer novel therapeutic approaches for atherosclerosis (Pi et al., 2019).

As we known, autophagy is considered to be an important self-protective mechanism for cell survival, with great potential in maintaining immune homeostasis and alleviating multiorgan failure under septic challenge (Ren et al., 2017). Since TFEB serves as a regulatory element in autophagic initiation, it reveals great potential in attenuating lethal septic response. In the mouse model of sepsis-induced cardiac dysfunction, TFEB-mediated autophagy showed obvious beneficial for migrating myocardial injury (Li F. et al., 2016).

In osteoarthritis (OA), the expression and nuclear transportation of TFEB presented with marked inhibition in both human and mouse chondrocytes (Zheng et al., 2018). It was reported that in the mouse OA model, TFEB overexpression by lentiviral transfection improved cartilage degradation and down-regulated chondrocyte apoptosis and senescence by enhancing autophagy, which might be a promising therapeutic candidate for OA treatment (Zheng et al., 2018). Accumulating evidences have indicated TFEB plays emerging role in the onset and development of other inflammatory diseases. For example, decreased TFEB expression through the phosphorylation by ERK increases inflammation and mitochondrial damage in the lung tissue and alveolar epithelial cells, contributing to the occurrence of pneumonia (Liu W. et al., 2019). Elevated phosphorylated level and cytoplasmic retention of TFEB lead to blocked lysosomal autophagy pathway and enhanced pancreatic proteasome activity, thus leading to pancreatitis (Wang et al., 2019). TFEB deficiency can also lead to the downregulation of lipoprotein ApoA1 which is linked to colitis susceptibility (Murano et al., 2017). Given that, TFEB maybe a new target for intervention and treatment of various inflammatory diseases.

### Neurodegenerative Diseases

One of the evident pathological features of neurodegenerative diseases is the accumulation of abnormal proteins, such as α-synuclein in Parkinson disease (PD), amyloid β (Aβ) and tau in Alzheimer disease (AD), huntingtin (HTT) in Huntington disease, polyglutamine-expanded androgen receptor (polyQ-AR) in X-linked spinal and bulbar muscular atrophy (SBMA), and mutant superoxide dismutase 1 (SOD1) in amyotrophic lateral sclerosis (ALS). These abnormal aggregates largely attribute to the impairment of autophagy-lysosomal function, which is reportedly closely associated with the activity of TFEB.

In a model of α-synuclein toxicity, excessive α-synuclein production in the nigral dopamine neurons was closely related to the decline of lysosomal function caused by cytoplasmic retention of TFEB (Decressac et al., 2013). While delayed activation of TFEB function through inhibition of mTORC1 blocked α-synuclein induced neurodegeneration and further disease progression (Decressac et al., 2013). In the mouse model of AD with tau spreading, exogenous TFEB expression was extensively noted in astrocytes, which was capable of capturing and degrading tau, thus markedly alleviating tau-associated pathological alterations (Martini-Stoica et al., 2018). In a mouse model of AD, the improvement of pathological changes could be augmented by exogenous supplementation of TFEB (Polito et al., 2014), which was achieved by inducing autophagy and reducing tau *via* promoting lysosomal exocytosis (Xu et al., 2020). The Aβ was degraded by enhanced lysosomal function *via* deacetylation of TFEB in microglia (Bao et al., 2016). In the mouse model of Huntington disease, the level of TFEB and the expression of its targeted gene were found with significant reduction. TFEB activation by restoration of PGC-1α is sufficient to reduce HTT aggregation and neurotoxicity (Tsunemi et al., 2012). Besides, trehalose was capable of activating TFEB by down-regulating AKT activity, thereby increasing the clearance of protein aggregations and reducing neuropathologic phenotypes (Palmieri et al., 2017a; Rusmini et al., 2019), while silencing TFEB significantly disturbed the pro-degradation activity of trehalose (Rusmini et al., 2019). SBMA is characterized by proximal muscle weakness due to the abnormal accumulation of polyQ-AR, which causes degeneration of the lower motor neurons in the spinal cord and brain stem (Cortes and La Spada, 2019). The activation of TFEB is noted in normal AR, but reveals interference by polyQ-AR, further impairing autophagy and promoting the pathogenesis of SBMA (Cortes et al., 2014). While TFEB overexpression by the upregulation of nuclear factor-YA markedly promoted the clearance of pathogenic AR protein in motor neurons and muscles of SBMA mouse model, thus mitigating the behavioral and pathological impairments (Tohnai et al., 2014). ALS is a motor neuron disorder characterized by abnormal accumulation of mutant SOD1 (Cortes and La Spada, 2019). Dysregulated TFEB localization was found in brain samples of ALS patients (Wang et al., 2016), while overexpression of TFEB by plasmid induced autophagy to clear accumulated SOD1, thus maintaining cell survival and proliferation (Chen et al., 2015). Therefore, TFEB is critically involved in the pathophysiological process of various neurodegenerative diseases as its key role in regulating lysosomal function, and maintaining TFEB activity can be considered as an effective way to accelerate the clearance of abnormally accumulated proteins.

## Conclusion and Perspective

With the deepening understanding of TFEB, its regulatory pathway, specific function, and relationship with the onset of multiple diseases have been uncovered, making TFEB an indispensable transcription factor for cellular homeostasis. Upstream regulators of TFEB have been well studied. Inhibitors including mTORC1, AKT, GSK3β, ERK2, and MAPK4 act mainly through the phosphorylation of TFEB. Activation of TFEB takes effects either by dephosphorization, such as calcineurin, or by inhibiting TFEB cascades, such as AMPK and PKC for down-regulation of mTORC1 and GSK3β, respectively. Kruppel-like factor 15 (KLF15) is another discovered TFEB activator that augments TFEB protein content and nuclear translocation in the cardiomyocyte (Trivedi et al., 2020). While the exact mechanism of KLF15 regulating TFEB still merits further investigation. Among the downstream TFEB effectors, the CLEAR elements, PPAR-α, and PGC-1α are the most studied. Regarding the regulation of TFEB activity, cytoplasmic retention and nuclear transfer have been extensively reported by quantities of studies, while the precise mechanism underlying nuclear export remains further elucidated. Although phosphorylation has been accepted as the main mechanism for TFEB activation, other modifications, like acetylation, need further clarification. Given the important role of TFEB in the pathogenesis of many human diseases, the current understanding of TFEB-related human diseases mainly focuses on its association with lysosome and autophagy, which has been identified involving in catabolic hyperactivity in tumors, imbalanced host responses in inflammatory diseases, and accumulation of abnormal aggregates in neurodegenerative diseases.

Therapies targeting TFEB might exert promising impacts on disease remission. With regard to the great heterogeneity among various malignancies, disparate strategies can be adopted under specific circumstances. For tumor cells of melanoma and multiple myeloma, the expression of TFEB is upregulated to present with anticancer effects *via* triggering autophagic cell death. Nevertheless, down-regulation of TFEB is needed to increase chemotherapy sensitivity for tumor cells with drug resistance. In inflammatory diseases, the pathological phenotypes can be relieved *via* upregulation of TFEB which is able to migrate oxidative stress in response to inflammatory imbalance and facilitate the clearance of inflammatory substances through autophagy. As for neurodegenerative diseases, the accumulation of pathogenic proteins due to lysosomal dysfunction can be alleviated by the upregulation of TFEB activity.

Currently, TFEB-related drugs have been developed. A synthesized curcumin derivative termed C1 enhances nuclear translocation of TFEB through specifically binding to the N-terminal of TFEB (Song et al., 2016), and it has been proved to ameliorate beta-amyloid precursor protein and tau pathology in Alzheimer’s disease models (Song et al., 2020). Other drugs, including resveratrol, progestin R5020, and 3,4-dimethoxychalcone are also reported to augment TFEB translocation into nucleus and enhance the function of TFEB (Chen et al., 2019; Tan et al., 2019a; Zhou et al., 2019). In addition, there are few reports with regard to TFEB inhibitors, e.g., Mn induces decreased nuclear localization of TFEB and results in autophagy dysfunction in astrocytes of mouse striatum (Zhang et al., 2020). Taken together, an understanding of the biological significance and pathophysiological roles of TFEB is favorable for further exploring novel yet efficient therapeutic measures for human diseases.

## Author Contributions

S-YZ and R-QY: literature investigation and data curation. S-YZ, R-QY, X-HD, CR, and Y-MY: writing-original draft, figure preparation, writing-review and editing. Y-XL and P-YZ: writing-review and editing. All authors contributed to the article and approved the submitted version.

## Conflict of Interest

The authors declare that the research was conducted in the absence of any commercial or financial relationships that could be construed as a potential conflict of interest.

## Publisher’s Note

All claims expressed in this article are solely those of the authors and do not necessarily represent those of their affiliated organizations, or those of the publisher, the editors and the reviewers. Any product that may be evaluated in this article, or claim that may be made by its manufacturer, is not guaranteed or endorsed by the publisher.
